# Characterization of *Rosculus vilicus* sp. nov., a rhizarian amoeba interacting with *Mycobacterium avium* subsp. *paratuberculosis*

**DOI:** 10.3389/fmicb.2023.1324985

**Published:** 2023-12-22

**Authors:** Amélie Jessu, Vincent Delafont, Jean-Louis Moyen, Franck Biet, Ascel Samba-Louaka, Yann Héchard

**Affiliations:** ^1^Université de Poitiers, CNRS, EBI, Poitiers, France; ^2^Laboratoire Départemental d’Analyse et de Recherche de la Dordogne, Coulounieix-Chamiers, France; ^3^Institut National de Recherche pour l’Agriculture, l’Alimentation et l’Environnement, Université de Tours, UMR 1282, Infectiologie et Santé Publique, Nouzilly, France

**Keywords:** amoeba, *Rosculus*, rhizarian, *Mycobacterium*, paratuberculosis, genome, protist, water

## Abstract

Free-living amoebae are described as potential reservoirs for pathogenic bacteria in the environment. It has been hypothesized that this might be the case for *Mycobacterium avium* subsp. *paratuberculosis*, the bacterium responsible for paratuberculosis. In a previous work, we isolated an amoeba from a water sample in the environment of infected cattle and showed that this amoeba was associated with *Mycobacterium avium* subsp. *paratuberculosis*. While a partial 18S rRNA gene has allowed us to suggest that this amoeba was *Rosculus*-like, at that time we were not able to sub-cultivate it. In the present study, we succeeded in cultivating this strain at 20–25°C. This amoeba is among the smallest (5–7 μm) described. The sequencing of the whole genome allowed us to extract the full 18S rRNA gene and propose this strain as a new species of the *Rosculus* genus, i.e., *R. vilicus*. Of note, the mitochondrial genome is particularly large (184,954 bp). Finally, we showed that this amoeba was able to phagocyte *Mycobacterium avium* subsp. *paratuberculosis* and that the bacterium was still observed within amoebae after at least 3 days. In conclusion, we characterized a new environmental amoeba species at the cellular and genome level that was able to interact with *Mycobacterium avium* subsp. *paratuberculosis*. As a result, *R. vilicus* is a potential candidate as environmental reservoir for *Mycobacterium avium* subsp. *paratuberculosis* but further experiments are needed to test this hypothesis.

## 1 Introduction

Free-living amoebae (FLA) are single-celled eukaryotes, omnipresent in water and soil environments (Rodríguez-Zaragoza, 1994; Samba-Louaka et al., 2019). These protists share common morphological features, notably pseudopods that allow them to move upon surfaces and to thrive by phagocytosis. Even though they are likely to play an important role in microbial ecology due to their ability to ingest bacteria as their main food sources (Jürgens and Matz, 2002), FLA have yet to be adequately described.

FLA constitutes a highly polyphyletic group of microbial eukaryotes and can be affiliated not only to Amoebozoa, the sole supergroup exclusively represented by amoeboid microorganisms, but also to Opisthokonta, Heterolobosea and Rhizaria. Among those three large eukaryotic groups, FLA consist mostly in small clades of amoebae, featuring a varied array of cell morphologies (Bass et al., 2009; Samba-Louaka et al., 2019). Among Rhizaria, the most notorious amoebae representatives are Vampyrellids, a group of predatory amoebae found in freshwater and marine environments. They can feed on bacteria and other eukaryotic preys (Hess and Suthaus, 2022). Additionally, other lesser known rhizarian amoebae were recently described. Most notably, molecular analyses highlighted the existence of a potentially very diverse clade, the Sainouroidea. The latter encompasses several genera of small-sized amoeba, such as *Rosculus*, which can be found in cattle, snake, fish feces, freshwater and soil (Schuler et al., 2018).

While FLA ingest bacteria by phagocytosis, some bacteria can resist phagocytosis and, consequently, might more specifically be able to resist phagocytosis by the immune cells of animals. This is due to a high level of conservation between phagocytosis mechanisms of FLA and animal cells (Escoll et al., 2013). This resistance was initially described for *Legionella pneumophila*, and many similar observations were made later on, involving diverse bacteria such as *Mycobacterium* spp., including *Mycobacterium avium* subsp. *paratuberculosis* (MAP) the bacterium responsible for paratuberculosis (Drancourt, 2014; Claeys and Robinson, 2018). In addition, amoeba-resistant bacteria might become more virulent insofar as they are adapted to the phagocyte environment and express virulence genes, thereby exemplifying the concept of coincidental evolution (Primm et al., 2004; Sun et al., 2018). Consequently, interest in FLA was revived by their capacity to become reservoirs of pathogenic bacteria in the environment.

It has been hypothesized that amoebae might be environmental reservoirs of Mycobacteria (Salah et al., 2009). To test this, we tried to isolate amoebae from water in the environment of infected cattle and look for the association between MAP, the bacterium responsible for paratuberculosis, and indigenous amoebae (Samba-Louaka et al., 2018). One amoeba was detected and identified, via 18S rRNA partial gene sequencing, as a *Rosculus*-like amoeba belonging to the supergroup Rhizaria. However, we were not able at that time to maintain the growth of this strain.

In this work, we ultimately identified optimal conditions for growing and isolating the strain associated with MAP. We fully sequenced the genome of this amoeba and analysis of the 18S rRNA gene sequence allowed us to confirm this amoeba among the *Rosculus* genus. Finally, an interaction study was performed to test the ability of this amoeba to phagocytose MAP.

## 2 Materials and methods

### 2.1 Isolation and cultivation of amoeba

This amoeba was previously isolated from an environmental water sample from the environment of infected cattle (Samba-Louaka et al., 2018). A sample (cryotube) frozen at that time was used to obtain a pure culture by critical dilution. Then, the cultivation was maintained within PAS buffer (Na_3_C_6_H_5_O_7_ 1 g/L, MgSO_4_:7H_2_O 0.4 M, CaCL_2_:2H_2_O 0.05 M, Na_2_HPO_4_:2H_2_O 0.25 M, KH_2_PO_4_ 0.25 M, pH 6.5) containing *E. coli* (5.10^8^ bacteria/mL) as nutrient.

To determine the optimal growing temperatures, *Rosculus vilicus* was cultured at 20, 25, 30 or 37°C for 24 h. Cells were detached and counted after an incubation of 3, 6 and 24 h. The experiment was repeated three times.

### 2.2 DNA extraction and genome sequencing

From pure culture, DNA was extracted in order to sequence this amoeba genome with two sequencing technologies, short and long-read approaches. Briefly, lysis buffer was added directly on adherent *R. vilicus*. Then, DNA was extracted with Blood & Tissue kit (Qiagen) following the manufacturer’s recommendations and quantified using the Qubit fluorometer (Thermo Fischer Scientific).

For the long-read approach, 3 μg of DNA was used for library preparation using the Oxford Nanopore Technologies ligation kit (SQK-LSK109) according to the manufacturer’s protocol with the Long Fragment Buffer (LFB). The library was then loaded on a flow cell (R9.4.1) and sequenced with MinION Mk1C. Base calling was done using Guppy v6.5.7 in high-accuracy mode. Simultaneously, genomic DNA was sent to SeqCenter (Pittsburgh, PA, USA) for library preparation and short read sequencing on an Illumina NovaSeq 6000. Demultiplexing, quality control and adapter trimming were performed with BCL convert software at SeqCenter.

### 2.3 Phylogenetic and genome analysis

We strived to follow a hybrid-based approach inspired by recently published protocols (Wick et al., 2023) to assemble a high-quality draft genome. For this, initial assembly was performed using Flye v. 2.9.1 (Kolmogorov et al., 2019) with long reads only, displaying quality score (Q) > 9 after base calling. A step of self-polishing was then implemented using Medaka v 1.7.2. Illumina reads were trimmed based on *Q* > 30 using Cutadapt v 4.4 (Martin, 2011), and for additional polishing of contigs using Polypolish v 0.5 (Wick and Holt, 2022). The resulting, fully polished assembly was then binned using Metabat2 V 2.15 (Kang et al., 2019). Manual inspections of bins were performed to ensure proper binning of sequences. For inferring phylogeny, Barrnap v 1.2.2 was used to identify and extract gene sequences coding for ribosomal RNA.^1^ Full-length 18S rRNA gene sequence was used to reconstruct phylogeny using IQ-TREE v 2.0.3 (Minh et al., 2020). The optimal substitution model was chosen based on the output of ModelFinder (Kalyaanamoorthy et al., 2017), according to the best Bayesian information criterion score. The robustness of nodes was tested by 1,000 iterations of conventional bootstraps and 1,000 iterations of the Shimodaira–Hasegawa approximate likelihood ratio test.

Mitochondrial genome was extracted based on joint analysis of graph assemblies, contig coverage and GC content. After manual and putative identification, the supposedly mitochondrial genome was further confirmed as such using multiple dedicated annotation tools, i.e., MITOS 2 (Donath et al., 2019), Mfannot^2^ and RNAweasel^3^ to identify mitochondrial proteins and RNAs. The nuclear genome and mitochondrial genome data were deposited at NCBI under the BioProject ID PRJNA1029052.

### 2.4 Morphological characterization of amoeba strain

Trophozoites and cyst-like forms from *R. vilicus* were observed by microscopy (Olympus IX73 inverted microscope, 1,000x magnification) using a differential interference contrast. All sizes were measured using ImageJ (Schneider et al., 2012). The morphological comparison between trophozoite and cyst-like forms was also assessed by electron microscopy. For scanning electron microscopy (SEM), cells were cryofixed in nitrogen with VCM (Leica), sublimed and 4 nm wolfram coated in ACE 600 (Leica). Each step was performed under vacuum and samples were transferred from one station to another using VCT 500 (Leica). Samples were observed at −100°C in scanning electron microscope Teneo VolumeScope (Thermo Fisher) to 3.5 kV and 10 kV.

For transmission electron microscopy (TEM), amoebae were fixed with 2.5% glutaraldehyde and 2% osmium tetroxide. Dehydration was carried out using different concentrations of acetone. Epon resin were used for impregnation and embedding process. After cutting with ultramicrotome UC6 (Leica), samples were contrasted with uranyl acetate and lead citrate. Samples observations were proceeded on transmission electron microscope (JEOL 1010) at 80 keV. Images were acquired with Quemesa camera (Olympus).

### 2.5 Infection experiments

To study the permissiveness of *R*. *vilicus* to MAP infection, an infection experiment was set up using a MAP K10-GFP strain (Li et al., 2005). *R. vilicus* (2.10^5^ cells) were added in each well of a 6-well plate in a 2 mL PAS buffer and incubated for 1 h at 20°C. MAP clumps were disaggregated with 3 passages in 27 gauges needle, centrifuged for 5 min at 200 *g* and added to *R*. *vilicus* at MOI 10 during 2 h at 20°C. Amikacin (100 μg/mL) was added for 2 h to remove extracellular mycobacteria before the washing step. Finally, the infected cells were incubated in PAS buffer added with *E. coli* (4.10^8^ bacteria/mL) for 3 days at 20°C. Colocalization of MAP K10-GFP with *R*. *vilicus* was observed under fluorescence microscope (Olympus IX51 inverted fluorescence microscope).

## 3 Results

### 3.1 *Rosculus vilicus* grows preferentially between 20 and 25°C

**Figure 1:**
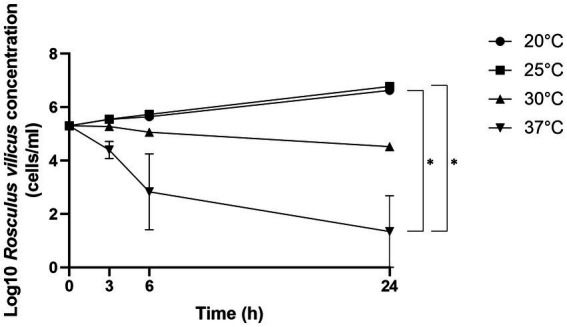
*R. vilicus* optimal growth was at 20°C and 25°C. Growth comparison of *R. vilicus* was tested by counting number of cells per ml at different incubation temperatures. The results represent three independent counts, and errors bars represent the standard error of the mean (±SEM). Statistical analysis was performed using Kruskal-Wallis test and uncorrected Dunn’s test (*p < 0.05).

We previously isolated a *Rosculus*-like amoeba that we cultured at 30°C but we ultimately lost over subcultures, suggesting at that time that growth conditions were not appropriate for this isolate. To determine the optimal temperature, we cultivated this amoeba at 20, 25, 30, and 37°C. As demonstrated in this study, the optimal growth temperatures ranged from 20°C to 25°C. Indeed, at 30°C the development was inhibited while at 37°C, the total number of cells decreased, suggesting cell death (Figure 1).

### 3.2 *Rosculus vilicus* shares morphological features with *Rosculus* genus

**Figure 2:**
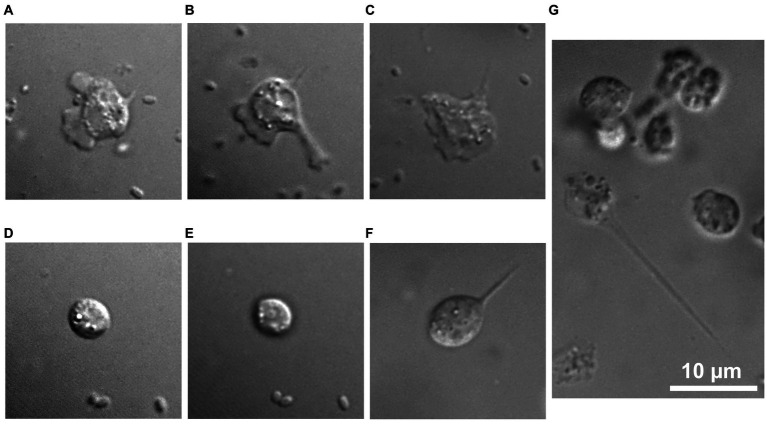
*R. vilicus* adopts different forms. Trophozoite forms **(A–C)**, rounded cells **(D,E)** and floating forms with a stem **(F,G)**. Bar length represents 10 μm.

**Figure 3:**
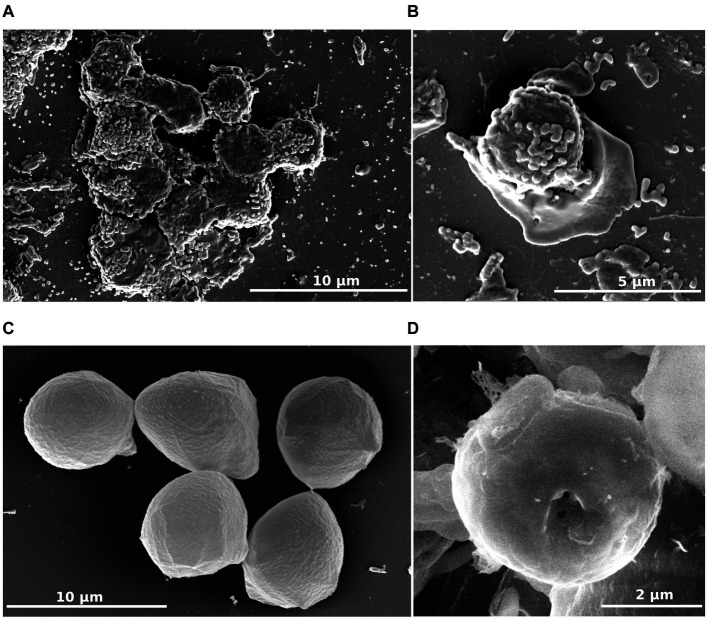
*R. vilicus* external morphology viewed by scanning electron microscopy. Trophozoite forms **(A,B)** and cyst-like forms **(C,D)**.

To characterize this isolate, microscopic studies were performed. Differential interferential contrast micrographs showed a monopodial lobose amoeba with a well-defined frontal hyaline area. Trophozoite cells usually displayed rapid eruptive locomotion with some lateral eruption (Figures 2A–C). An uroidal structure could be observed on some trophozoites, the opposite of that found in the hyaline area (Figures 2A–C). Average length was estimated at 7.1 μm (range of 4.2–13.8 μm) and average width was 4.8 μm (range of 2.7–12.1 μm). During observations, some amoebae adopted a floating form, more rounded than trophozoite (6.0 μm average length and 4.9 μm average width), harboring a long stem, about 18.9 μm on average (10.2–31.4 μm) (Figures 2F,G). All amoeba cells contained a large vacuole and several granularities. Given the ability of amoebae to differentiate into cyst forms, we incubated our amoebae in an encysting buffer for 4 days. We observed rounded cells with an average width of 4.8 μm (Figures 2D,E). The observation of trophozoites and cyst-like forms under a scanning electron microscope confirmed their estimated size (Figures 3A–D). The cell surface of cyst-like forms displayed one ostiole (Figure 3D).

**Figure 4:**
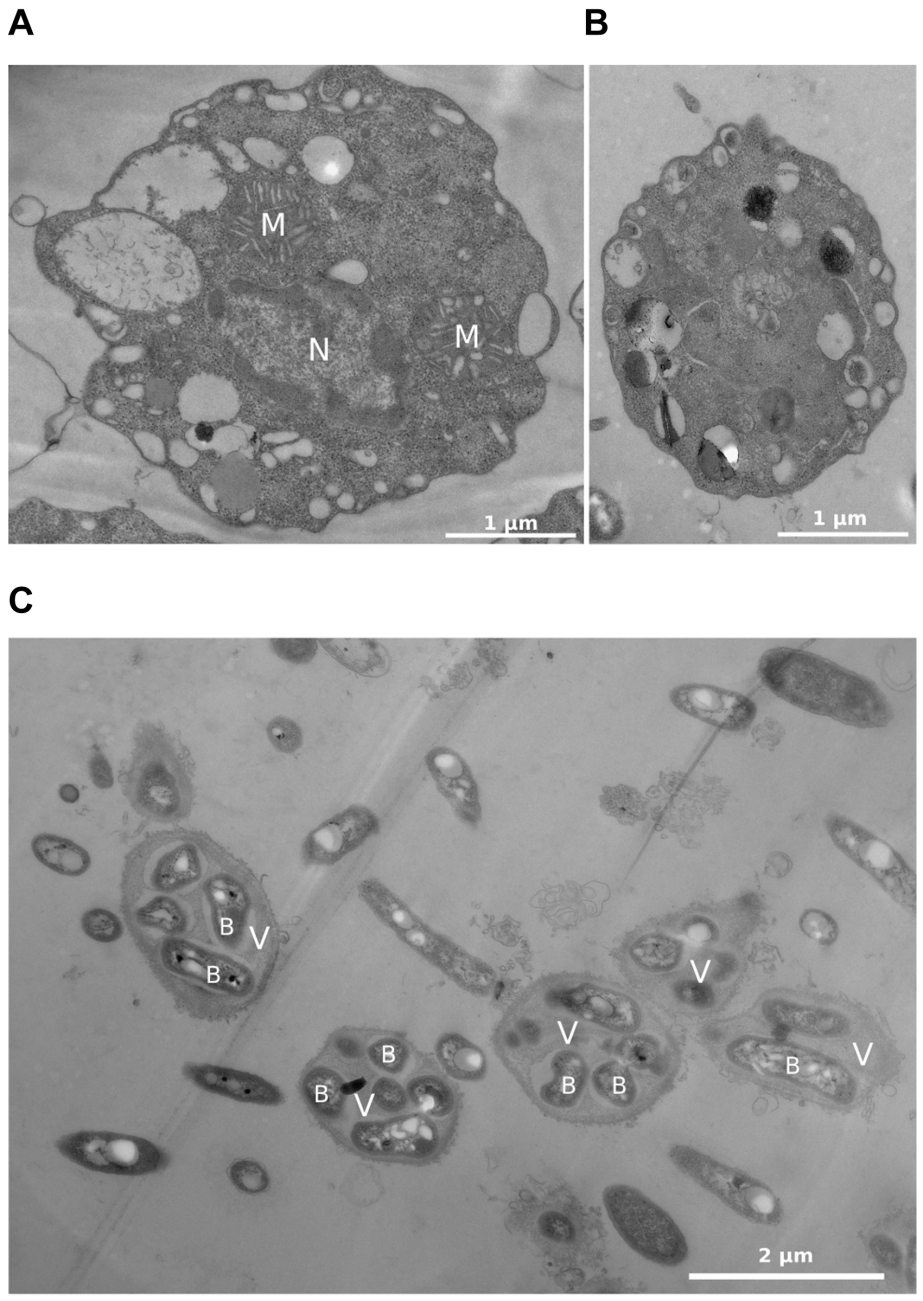
*R. vilicus* internal ultrastructure viewed by transmission electron microscopy; trophozoite form **(A)** cyst-like form **(B)** and extracellular medium observed for encystment condition containing vesicles and bacteria **(C)**. N, nucleus; M, mitochondria; V, vesicle containing bacteria; B, bacteria.

Transmission electron micrographs enabled us to observe the ultrastructure of both trophozoites and cyst-like forms. The cell membrane in both trophozoites and cyst-like forms consisted of a thin single membrane (Figures 4A,B). Regarding trophozoites, the cytoplasm contained a large nucleus bordered by a dense chromatin, large mitochondria with discoidal mitochondrial cristae and several vacuoles (Figure 4A). Interestingly, in encystment conditions, the extracellular medium contained free bacteria and numerous vesicle-like structures filled with bacteria (Figure 4C).

### 3.3 *Rosculus vilicus* is a new species with a large mitochondrial genome

As no genome was described in the *Rosculus* genus and even in the Sainouroidea clade, we decided to further characterize the isolate on this aspect. Whole genome sequencing was undertaken, using a hybrid, short and long-read sequencing approach. Based on this, the assembled genome of this isolate was predicted to be 40.8 Mb long, showing 37.7% GC enrichment (Table 1). While *E. coli* genome (used as a food source) was recovered in the sequencing data, no other bacterial genome could be retrieved. In this hybrid assembly, particular focus was given to recovery of the mitochondrial genome. One contiguous, highly covered sequence was recovered from the assembly, consisting in a 184,954 bp long sequence, with overall 24.9% GC enrichment. While it was unusually long, even when compared with other mitochondrial genomes from other related protists from the Cercozoa group (Wideman et al., 2020) we are confident that it corresponds to a *bona fide* mitochondrion. Indeed, mitochondrial genome annotation revealed gene repertoires similar to mitochondria from other cercozoans, while showing even coverage and reproducible assembly features, even with short reads only (Supplementary Figure S1).

**Table 1:** Statistics and features of *R. vilicus* nuclear and mitochondrial genome assemblies.

	*Rosculus vilicus* nuclear genome	*Rosculus vilicus* mitochondrial genome
Total length	40,828,467	184,954
Coverage	309X (Illumina) / 300X (MinIon)	1,152X (Illumina) / 1,110X (MinIon)
GC content	37.72%	24.95%
No of contigs	69	1
Largest contig	4,194,092	184,954
N50	1,847,826	184,954
N90	622,946	184,954
L50	8	1
L90	22	1

This sequencing effort brought into focus the first draft genome for the whole Sainouroidea. Because of this, however, comparative analyses of gene and genome sequences was clearly hampered, and consequently restricted to widely used marker genes. Given the resulting paucity of genomic information on this clade (and on Cercozoa in general), in order to investigate the taxonomy and phylogenetic positioning of our isolate, we focused on 18S rRNA gene sequences. The full-length 18S rRNA gene sequence (2,128 bp) used for BLASTn search showed its highest match with a sequence designated as ‘uncultured Cercozoa isolate 1’ (98.18% identity, 59% of query coverage). This sequence indeed corresponds to the previously published partial 18S rRNA gene sequence of this isolate (Samba-Louaka et al., 2018).

**Figure 5:**
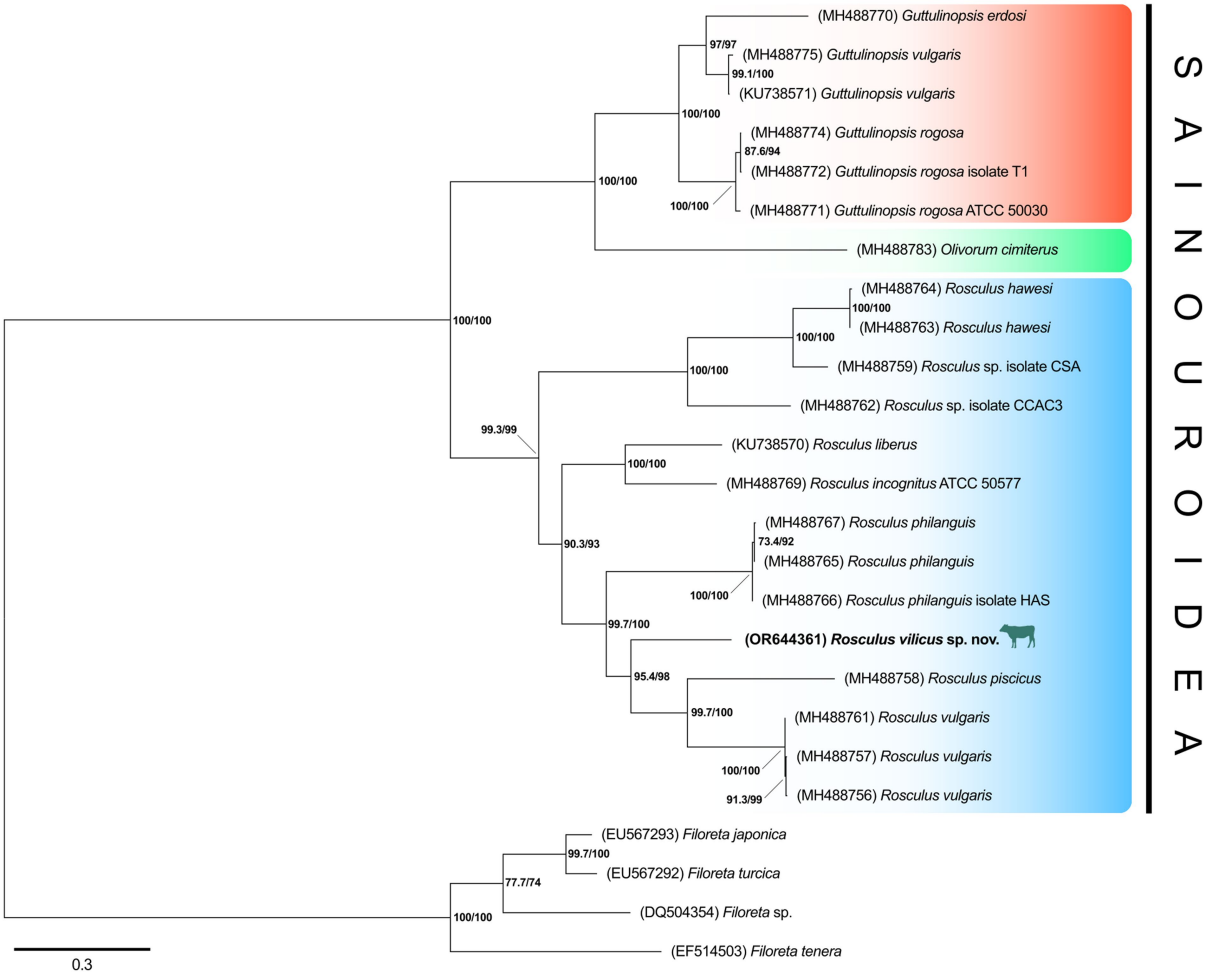
*R. vilicus* is likely a new species, closely related to *R. vulgaris*. Phylogeny of Sainouroidea rooted with Filoreta as outgroup based on 18S rRNA gene.

To confirm the belonging of this amoeba to the *Rosculus* genus, a phylogenetic tree of the 18S rRNA gene was constructed, using a set of rRNA sequences from all available Sainouroidea representatives as of June 2023. Phylogeny inferences clearly placed our isolate within the *Rosculus* genus (Figure 5). The relationship between the sequence from our isolate, compared to other known sequences of the *Rosculus* genus, justified our proposal of a new species, which we tentatively named *Rosculus vilicus* sp. *nov*., pertaining to the farm where the amoeba was isolated.

### 3.4 *Rosculus vilicus* interacts with MAP for at least a few days

**Figure 6:**
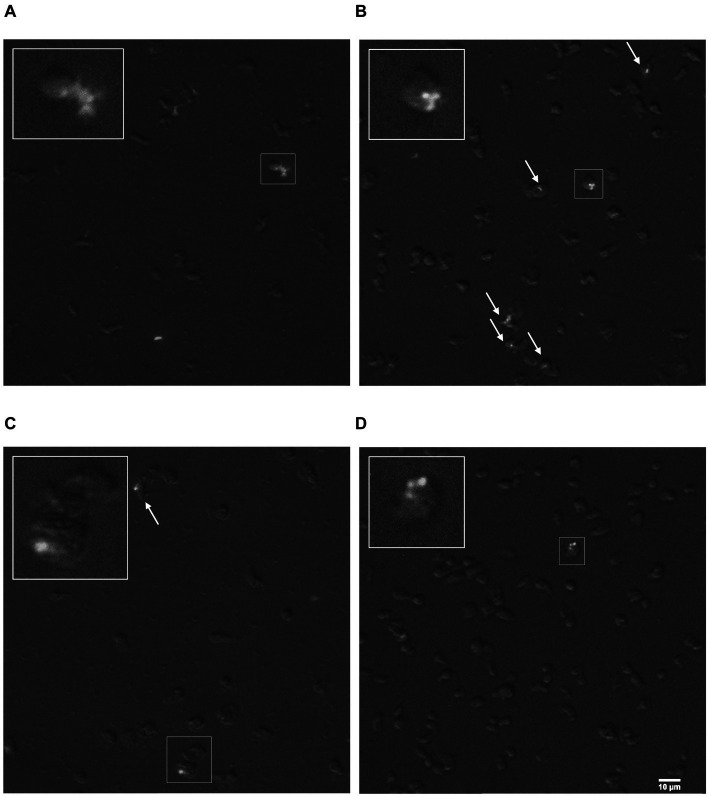
*R. vilicus* co-localized with MAP K10-GFP. Micrographs with magnification x400 show *R. vilicus* in co-localization with MAP K10-GFP at 4 h **(A)** 24 h **(B)** 48 h **(C)** and 72 h post-infection **(D)**. The interaction is highlighted with white arrows or framed and magnified three times.

*Rosculus vilicus* was previously isolated in association with a MAP (Samba-Louaka et al., 2018). Here, we evaluated the permissiveness of *R. vilicus* to the MAP K10 strain. An interaction experiment was carried out with the MAP K10-GFP strain to follow the fate of the bacteria by fluorescence microscopy. The result showed that *R. vilicus* co-localized with MAP K10-GFP at least 72 h post-infection (indicated by white arrows, Figures 6A–D). SomeMAP K10 were localized extracellularly (Figures 6A,B). Furthermore, video-microscopy experiments highlighted *R. vilicus* moving by ameboid movement with MAP K10 following the same course (Supplementary Figures S2, S3). These results suggest an association for a period of at least 3 days between *R. vilicus* and MAP.

## 4 Discussion

It is well-known that FLA can host pathogenic and non-pathogenic bacteria, thereby playing a potential role on their persistence in the environment. However, in the environment the interactions between FLA and MAP have been very poorly investigated (Drancourt, 2014).

In a previous study, aimed at isolating FLA associated with MAP in the environment of infected cattle, we isolated a strain, which we identified at the time as being somehow related to *Rosculus*, from a drinking trough (Samba-Louaka et al., 2018). This strain was grown for a short period of time, but lost along subcultures, most likely indicating that the growth conditions were not fully adequate at that time. Here, the first challenge was to identify the conditions for optimal and perennial cultivation of this FLA. Different incubation temperatures were tested, clearly showing an optimum at 20–25°C. This result is in accordance with the cultivation conditions described for other *Rosculus* species (Schuler et al., 2018). Current knowledge on Sainouroidea, and more particularly on *Rosculus* spp., suggests that they are amphizoic (Bass et al., 2016). Members of the *Rosculus* genus were repeatedly isolated from both soil and fecal matter (Schuler et al., 2018) and sometimes even directly from rectal sampling of the animal host (Hawes, 1955). This would indicate that *Rosculus* are not only able to grow in the environment, but also in the feces and even in the digestive system of their respective hosts. In the case of *Rosculus vilicus*, our inability to grow at 37°C may be surprising, as one may expect its ability to thrive at temperatures close to the bovine digestive system (Burfeind et al., 2010). The presence of complex bacterial consortia and oxygenation levels may play an important role in growth capabilities of this amoeba and would necessitate further characterization. Also, it is possible that *Rosculus* is present in feces but may be able to grow only in the environment when the temperature drops to 20–25°C and not in the gut at 37°C.

The morphology of *R. vilicus*, observed under light microscopy, displays features comparable to the *Rosculus* strain previously described in the literature. For example, its small size is a morphological trait found in the other *Rosculus* isolates described. In line with this, we observed that *R. vilicus* trophozoites were on average 7.1 μm long and 4.8 μm wide, while cyst-like forms were round and on average 4.8 μm in diameter. These observations correspond to the measurements of previously isolated *Rosculus* strains. Depending on the species, they are between 2.5 and 13 μm long and between 4.2 and 5.5 μm wide in their locomotive forms (Hawes, 1963; Bass et al., 2009; Tyml and Dyková, 2018). Trophozoite forms of *Rosculus* were shown to have a rapid eruptive locomotion with an elongate hyaline pseudopod. Endoplasm is described as crowded with vacuoles, resulting in a “sandy appearance” (Hawes, 1963; Bass et al., 2009). In our observations, similar morphological traits were noticed; rapid eruptive locomotion with one hyaline pseudopod and dense granuloplasm. No flagella were observed on *R. vilicus*, as described on the other *Rosculus* strains (Hawes, 1963; Bass et al., 2009; Tyml and Dyková, 2018). However, a new morphological trait, hitherto undescribed in *Rosculus* species, was a long stem on floating forms (Figures 3A,B). To our knowledge, it had not been described previously, and while the associated function is not obvious, we may hypothesize that adopting such a morphology may impact cell buoyancy, thereby facilitating dispersal along with the flow of liquid.

Cyst-like forms observed under light microscopy were round or oval-shaped, with refringent wall, and correspond to previously observed cystic form. Observation by TEM was more difficult because few cells were observed in our encystment conditions. The cells observed had a thin cell wall, contrary to what had been observed before, and which is generally found in amoeba encystment forms (Hawes, 1963; Bass et al., 2009; Tyml and Dyková, 2018). Despite the lack of thick cell wall, the observation of an ostiole with SEM suggests that it is really a cyst form.

As is the case with most protist clades, genomic information on FLA is dramatically lacking. This should stimulate a systemic attempt at producing genomic data for novel protists isolates (Sibbald and Archibald, 2017). In line with this intent, we proceeded with the genomic characterization of *R. vilicus*, which has yielded the first genomic data for the Sainouroidea and adds to the (poorly) known genomic diversity of rhizarian protists. The draft genome of *R. vilicus*, assembled through a combination of long and short read data, provided an estimate of its size, which is *ca.* 40.8 Mbp long. This genome size is comparable with other FLA from distinct groups, such as *Acanthamoeba castellanii* (43.8 Mbp; Matthey-Doret et al., 2022), *Vermamoeba vermiformis* (39.5 Mbp; Delafont et al., 2021) or *Naegleria gruberi* (41 Mbp, Fritz-Laylin et al., 2010). Further studies providing transcriptomic data on *R. vilicus* could provide an initial annotation of this genome, a prerequisite for in-depth studies of gene repertoire. Aside from this nuclear genome, our hybrid assembly approach enabled us identify and isolate the mitochondrial genome of *R. vilicus*, which is unusually long (185 kbp). This size estimate far exceeds the size of other mitochondrial genomes from the Cercozoa, for which recent estimates indicated sizes around 40 to 50 kbp (Wideman et al., 2020). While significantly larger than other known sequences from the same clade, mitochondrial gene repertoire of *R. vilicus* is highly comparable to that of other cercozoans and seems to once again prove that we have identified and correctly assembled this organellar genome (Supplementary Figure S1). To our knowledge, the characterization of *R. vilicus* mitochondrion represents the largest known mitochondrial genome ever described for a single-celled organism, thereby providing an interesting model for investigation of the dynamics of mitochondrial genome evolution. The novelty of this isolate should be seen as another argument in favor of the systematic implementation of genome sequencing for all novel protist isolates, as it represents a huge source of original findings.

In the process of assembling *R. vilicus* genome, we noted that no bacteria (apart from *E. coli* used as food source) was recovered. This suggests that *R. vilicus* does not live in symbiotic associations with other bacteria, as is the case for numerous other FLA (Samba-Louaka et al., 2019). However, one cannot rule out the possibility that the growth conditions we used, and more globally a switch from environmental to *in vitro* conditions, may have contributed to a loss of this association, on which we may have missed out in this study.

The strain isolated from water trough of infected cattle herds with paratuberculosis was a good candidate to verify our hypothesis that free-living amoebae could be MAP vectors. Since the first evidence the ability of mycobacteria species to survive in amoebae (Prasad and Gupta, 1978) different *Mycobacterium* species have been investigated regarding their interactions with phagocytic protozoan such as *Acanthamoeba*, *Dictyostelium* or *Tetrahymena*. Several *Mycobacterium* species were able to resist amoebal digestion, suggesting a role of amoebae in mycobacterial infections (Thomas and McDonnell, 2007). Most of these findings documented the involvement of amoebozoans and heteroloboseans representative in such interactions (Balczun and Scheid, 2017; Shi et al., 2021). However, recent work has demonstrated that numerous rhizarian FLA could also bear intracellular bacteria, thereby confirming that tight FLA-bacteria interactions indeed occur in all these taxonomic groups (Pohl et al., 2021; Solbach et al., 2021).

Few studies have related MAP association with amoebae. Among them, it was shown that incubation of MAP with *Acanthamoeba polyphaga* and *Acanthamoeba castellanii*, led to a decreasing MAP number in the first few post-internalization days, but increased after an extended period of several weeks (Mura et al., 2006; Whan et al., 2006; Phillips et al., 2020). Our results provide evidence that MAP can also persist for at least for 3 days in association with *R. vilicus. However, it was a rare event, as only a few amoebae were associated with the MAP K10-GFP and it was not possible to distinguish if the bacteria were inside (phagocytized) or outside the amoebae*. This suggests, added to previous observations using different FLA, that MAP might interact with a wide range of amoebae distantly related.

In conclusion, our study enabled to recover *R. vilicus*, a new species of *Rosculus* that grows preferentially between 20 and 25°C. Microscopic observations showed a small amoeba with morphological traits representative of the genus *Rosculus*. Whole genome sequencing added genomic data to the poorly known genomic diversity of rhizarian protists. Unexpectedly, this work highlighted the particularities mitochondrial genome size. *R. vilicus* can interact with MAP for at least a few days. This result suggests the possible role of amoebae in the persistence of MAP and contributes new evidence about the protists range that interact with MAP in environment.

### 4.1 Description of *Rosculus vilicus* sp. nov

Description of *Rosculus vilicus* [vi.li.cus. N.L. adj.] sp. nov.

Taxonomic assignment: Eukaryota; TSAR; Rhizaria; Cercozoa; Sainouroidea; Guttulinopsidae; *Rosculus*.

Diagnosis: Cercozoan, limax-shaped amoeba. Single nucleus. Found in freshwater. Trophozoites: 4.2–13.8 μm (average: 7.1 μm) in length and 2.7–12.1 μm (average: 4.8 μm) in width. Trophozoites can adopt a more rounded floating form which typically displays a 10.2–31.4 μm stem (average: 18.9 μm). The amoebae can form a cyst-like structure when starved (average: 4.8 μm). The amoebae feed on the bacteria *Escherichia coli*.

Type strain: Isolate AJ1 (available upon request). Isolated from cattle drinking trough located in Indre et Loire department, France. The amoebae were isolated on a non-nutrient agar plate seeded with *E. coli* and routinely cultivated on PAS buffer with *E. coli* (OD 0.5) at 20°C.

Etymology: vilicus from *villa* (“country house”) + *icus*; pertaining to the farm.

The genome size of this isolate is was predicted to be 40.8 Mb long, showing 37.7% GC enrichment. This is the first genome of *Rosculus* spp. and there is no genome available in related species shown in Figure 5. The sequence was deposited at NCBI under the BioProject ID PRJNA1029052.

## Data availability statement

The data presented in this study are deposited in the NCBI database under accession numbers OR644361 and PRJNA1029052.

## Author contributions

AJ: Conceptualization, Data curation, Writing – original draft, Writing – review & editing, Formal analysis, Investigation. VD: Writing – original draft, Writing – review & editing, Conceptualization, Data curation, Methodology, Formal analysis. J-LM: Writing – review & editing, Project administration, Funding acquisition. FB: Writing – review & editing, Conceptualization, Funding acquisition. AS-L: Writing – original draft, Writing – review & editing, Conceptualization, Data curation, Methodology, Formal analysis. YH: Conceptualization, Data curation, Funding acquisition, Supervision, Writing – original draft, Writing – review & editing.

## Footnotes

^1^ https://github.com/tseemann/barrnap

^2^ https://megasun.bch.umontreal.ca/apps/mfannot/

^3^ https://megasun.bch.umontreal.ca/apps/rnaweasel/

## Supplementary material

Supplementary Figure S1 The unusually large mitochondrion of *R. vilicus* shows a gene repertoire comparable to, and phylogenetic affinities with, other cercozoans. Analyses of mitochondrial contig coverage suggests that its unusual size does not result from artefactual assembly **(A)**. Gene and transfer RNA repertoire from *R. vilicus* almost perfectly mirror that of other cercozoans, as identified by Wideman et al. (2020). For tRNA, a red-filled square indicates an independent codon reassignment. The ‘extras’ category indicates the unusual codons N (auu) in A (uau) in B **(B)**. Maximum likelihood phylogeny of the COX1 protein (332 amino acid residues) shows clear affiliation of *R. vilicus* with other cercozoans, congruent with nuclear rRNA phylogeny **(C)**.

Supplementary Figure S2 *R. vilicus* and MAP K10-GFP co-localize on the video taken 4 hours post-infection.

Supplementary Figure S3 *R. vilicus* and MAP K10-GFP co-localize on the video taken 72 hours post-infection.
