# Supplementary figures and images for: Characterization of Rosculus vilicus sp. nov., a rhizarian amoeba interacting with Mycobacterium avium subsp. paratuberculosis
Source: Front Microbiol. 2023 Dec 22;14:1324985. doi: 10.3389/fmicb.2023.1324985 (PMC10770858; doi:10.3389/fmicb.2023.1324985)

a

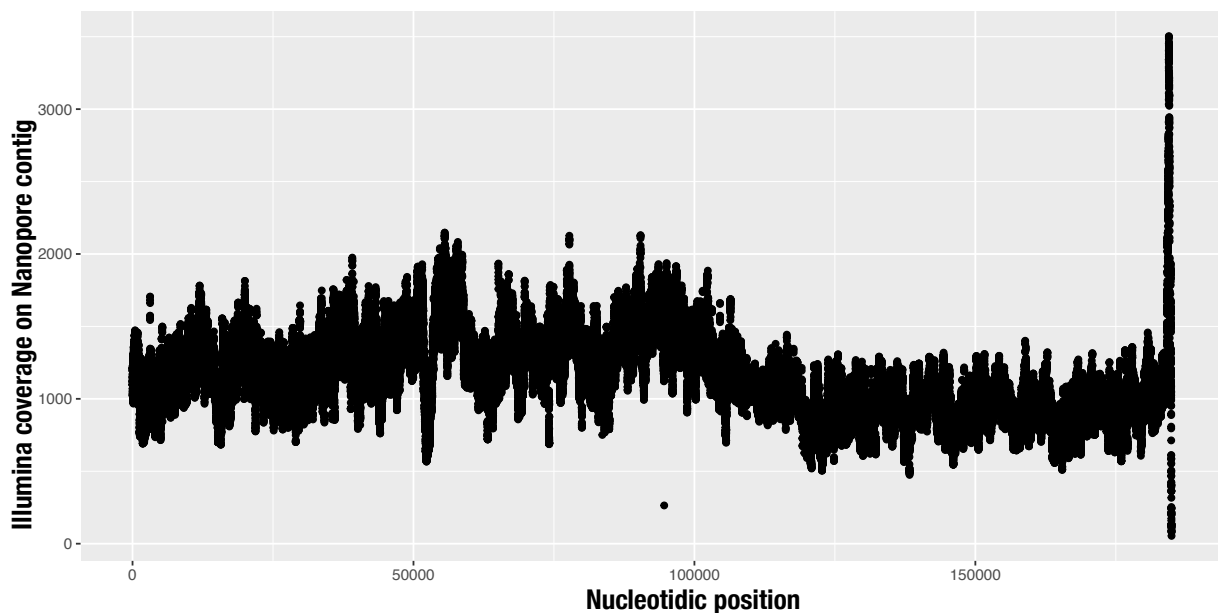

b

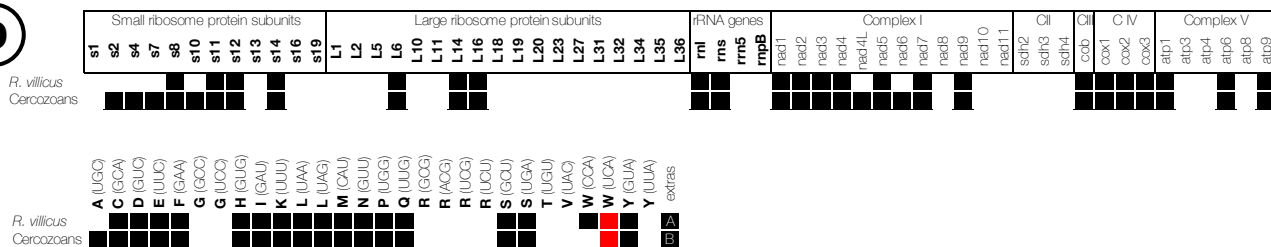

c

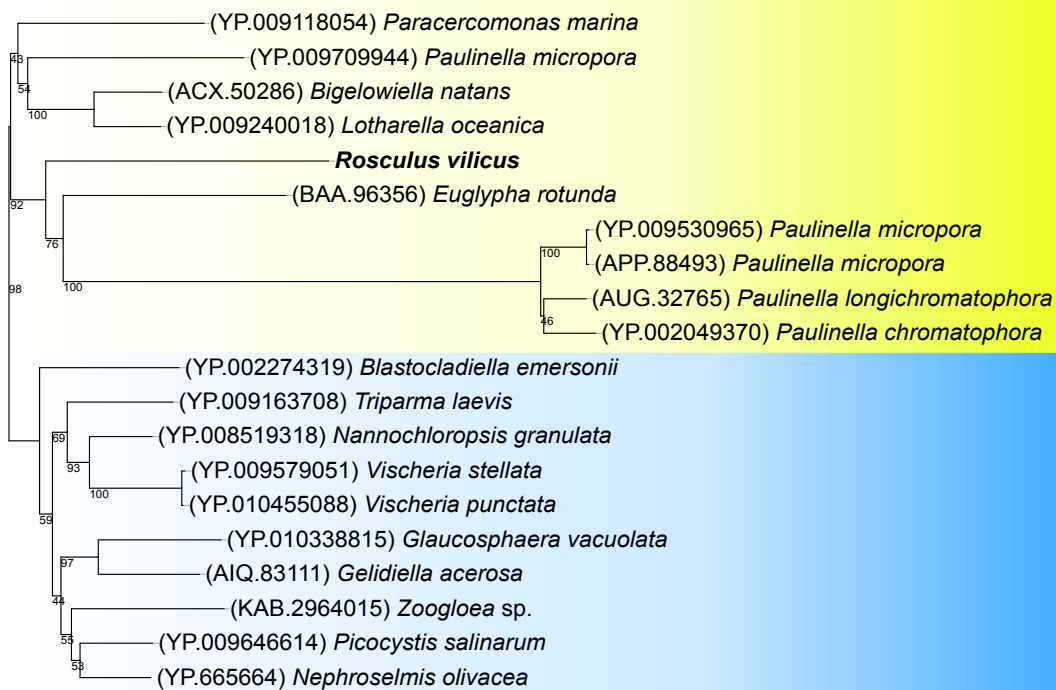

Cerczoza

TSAR

Tree scale: 0.1

Supplement: Supplementary file 3 [file Data_Sheet_1.PDF]
